# Utilizing a balloon sheath and miniprobe for diagnostic endoscopic ultrasound in eosinophilic esophagitis: a case series

**DOI:** 10.1186/s13089-024-00380-7

**Published:** 2024-08-21

**Authors:** Simon S. Rabinowitz, Rheu Candava, Blair Kady, Dalia Arostegui, Evan Grossman

**Affiliations:** 1 Pediatric Gastroenterology Division, Downstate Health Sciences University, 450 Clarkson Ave, Box 49, Brooklyn, NY 11203 USA; 2 Division of Gastroenterology and Hepatology, Department of Medicine, Downstate Health Sciences University, 450 Clarkson Ave, Box 49, Brooklyn, NY 11203 USA

**Keywords:** Endoscopic ultrasound, Miniprobe, Balloon sheath, Eosinophilic esophagitis, Esophageal remodeling, Subepithelial pathology, POCUS

## Abstract

**Background:**

Endoscopic ultrasound (EUS) is a unique example of POCUS, which allows the gastroenterologist to discuss subepithelial pathology immediately after an endoscopy. The challenges that are encountered to create an acoustic interface by adding free water during the endoscopy may be curtailing the full utilization of EUS during endoscopic procedures. Eosinophilic esophagitis (EoE) is a progressive inflammatory condition whose morbidity is related to esophageal wall remodeling. However, in clinical practice, in clinical guidelines, and in many trials, EoE outcomes are based on esophageal eosinophilia and symptoms. Hence, a method to identify and quantitate the thickening of the esophageal wall, could contribute to the management of this disease.

**Results:**

A modification of the approach employed to perform EUS during bronchoscopy was developed. An EUS miniprobe was positioned inside of a water filled balloon sheath. This technique permitted rapid and reproducible images acquisition of the total esophageal wall and its sublayers (mucosa, and submucosa + submucosa, which permitted derivation of the muscle layer). The presented series describes the results from  22 consecutive EoE patients. A full set of measurements from both the mid and distal esophagus were achieved in all EoE patients in an average time of less than 10 minutes.

**Conclusions:**

This pilot study supports further investigations evaluating this economical, convenient, and safe technique to follow EoE patients. In addition, this approach could be potentially employed in all patients who are found to have subepithelial gastrointestinal pathology during routine endoscopic procedures.

**Supplementary Information:**

The online version contains supplementary material available at 10.1186/s13089-024-00380-7.

## Background

Endoscopic ultrasound (EUS) is felt to have an essential and increasing role in upper gastrointestinal endoscopy (EGD) [1, 2], but only 1% of all endoscopies and fewer colonoscopies, include EUS [3]. EUS can be considered a unique form of point of care ultrasound (POCUS) since the gastroenterologist managing the patient personally acquires the images utilized in their care. POCUS is being frequently employed to quickly elucidate spatial relationships that can then guide interventions such as central line placement and trauma victim triage. Similarly, EUS is presently employed to drain fluid collections and to target tissue acquisition. The scope of POCUS to characterize anatomic features that aid in diagnosis is increasing into new domains such as emergency room POCUS echocardiography [4], and outpatient evaluation of intestinal wall thickening in inflammatory bowel disease patients [5]. EUS is similarly capable of adding an immediate three-dimensional appreciation of any subepithelial pathology which is identified during routine endoscopic procedures.

Conventional EUS, is most frequently performed with a dedicated, usually 5–12 MHz, echoendoscope in which  the probe is directly incorporated into a thicker scope. Alternatively, EUS can be performed with an ancillary miniprobe (EUS-MP) that passes through the biopsy channel of a standard endoscope or colonoscope. Thus, EUS-MP potentially has wider applications, reaching areas of the gastrointestinal tract that are only accessible to a narrower scope [6] and could complement any endoscopic procedure. Miniprobes are available with 12 or 20 MHz frequency. The 20 MHz probe provides higher resolution imaging and can yield more anatomical details. While the higher frequency sensor simultaneously limits penetration to 15–20 mm [7], this still permits imaging of the full thickness of almost any area reached by the endosonographer. As with all ultrasound, EUS-MP requires an acoustic coupling which is traditionally created by introducing a water/tissue interface. This can be challenging to maintain in areas bordering large lumens or immediately proximal to sphincters. In addition, adding variable amounts of free water could stretch the wall differentially, potentially compromising reproducibility of EUS measurements [8].

EUS-MP was initially employed to evaluate EoE in a seminal study in 2003 which demonstrated that 11 pediatric EoE patients had thickened esophageal walls compared to 8 controls [9]. A second group confirmed this relationship in adults with EUS utilizing an echoendoscope [10]. The widening that was described is now generally recognized as esophageal remodeling, the essence of EoE pathogenesis [11]. At the present time esophageal eosinophilia and symptoms are employed to follow the progress of EoE which does not correlate with wall thickening. A recent single center study employed serial EUS exams to demonstrate thickening of the esophageal wall in a cohort of adults with EoE who had no changes in their tissue eosinophilia, suggesting that incorporating this modality could identify EoE patients with a more aggressive form of EoE [12]. However, despite the important role of remodeling in EoE, EUS has not emerged as a valuable tool to explore this phenomenon or to guide patient care.

One potential explanation could be that filling the lumen of the gut with water to maintain the acoustic interface has frustrated endosonographers. A recent series of 1598 EUS cases to evaluate gastric wall cancer reported that the procedure required 300–800 ml of water added to the stomach [13]. An alternative approach is to place the miniprobe inside of a latex balloon sheath, similar to the technique employed in bronchoscopy. The sheath is pre-filled with water before the procedure, and the probe is then positioned against any section the sonographer wishes to image. This eliminates the need for infusion of free water in a sedated patient and permits the endosonographer to capture multiple images faster.

The present series was part of a larger study evaluating a role for EUS-MP in recognizing and monitoring esophageal remodeling in children with eosinophilic esophagitis (EoE). EUS established that esophageal wall thickening is associated with active EoE  in older teen agers and young adults and with appropriate therapy it  could be reversed [14]. This paper describes our experience employing a latex balloon and a miniprobe to perform 22 consecutive comprehensive EUS characterizations of the total and individual layer thicknesses in the mid and distal esophageal walls. A minimum of 12 measurements were obtained for each case and required an average time of less than 8.5 min.

## Methods

### Setting

Two hospitals with an established pediatric gastroenterology referral base and fellowship training program, which are part of the same inner-city medical school, Downstate Health Sciences University.

### Patients

The series consisted of 22 consecutive EUS cases (16 males, mean age 15 year) performed between 2019 and 2021. All participants over 12 years had informed consent and parents also signed  for those under 18 years. The consent/assent was for participation in an IRB approved study that included routine EUS during EGD performed to diagnose or monitor EoE. Fifteen patients had EoE, of which seven had active disease, (mean number of eosinophils/high power field: 33 distal and 12 mid esophagus) and eight were in remission. Seven patients were being screened for possible EoE, but on endoscopy lacked esophageal eosinophilia or endoscopic features specific for EoE.

### EUS

After a standard screening upper endoscopy was performed, a pre-assembled Olympus ultrasound miniprobe (UM-BS20-26R) inside of an Olympus latex balloon sheath (MAJ-643R) filled with water, was advanced through the 2.8 mm working channel of a GIF-Q180 or GIF-160 standard Olympus endoscope into the distal esophagus. Ultrasonography was performed using acoustic coupling from the water in the latex balloon sheath, obviating the need for adding free water or suctioning the air out of the esophagus. After obtaining the required images, the scope and balloon probe were positioned in the mid esophagus for the second set of measurements. Visualization was always maintained by changing the position of the assembly as required. Bringing the water filled balloon sheath adjacent to the esophageal wall created the interface without exerting unnecessary pressure. The balloon probe was easily moved to any portion of the lumen to quickly obtain confirmatory or additional measurements. An in-house video is available on a link in the supplementary material to illustrate the procedure.

**Fig. 1:**
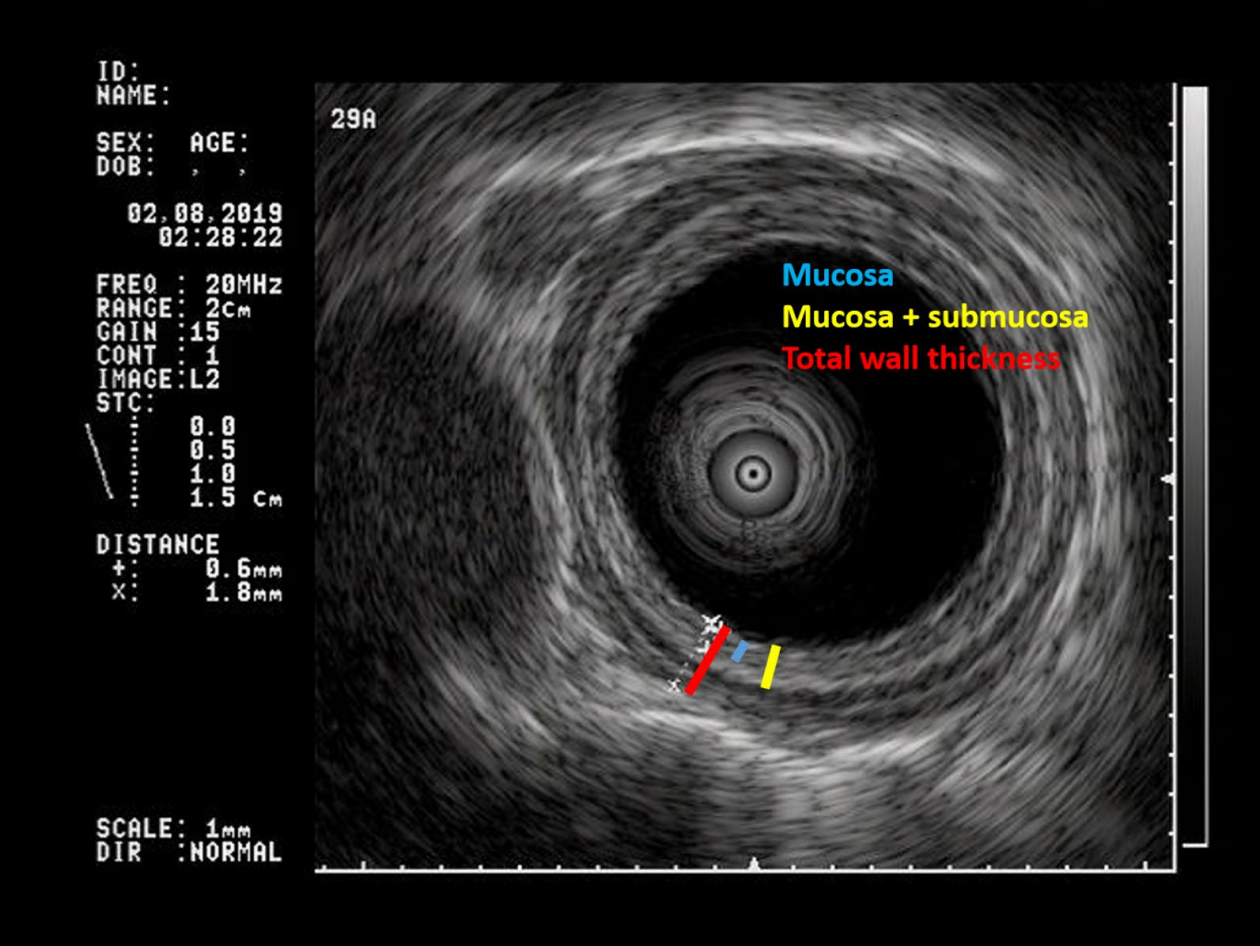
Endoscopic ultrasound images obtained using a miniprobe inside of a water filled latex balloon sheath to provide acoustic coupling in an 11-year-old boy. The blue bar defines the mucosa. The yellow bar defines the mucosa and submucosa. The red bar defines the full thickness of the esophageal wall. In our protocol, the muscle layer is derived by subtracting the yellow bar (mucosa plus submucosa) measurement from the red bar, the full esophageal wall thickness. In the figure, the thin hyperechoic band in the muscularis (beyond the yellow bar but within the red bar) represents the border between the circular and longitudinal muscles

Following the previously described protocol [15], each EUS study provided at least two sets of 3 measurments: (a) the mucosa; (b) the mucosa + submucosa; and (c) the total wall thickness (TWT) in both the mid and distal esophagus, (hence, 6 from both sites, to yield 12 measurements minimum per patient). The figure illustrates representative images. The study protocol required two values of TWT from each site to have < 20% variation, to minimize potential artifact and to maximize reproducibility. If the first two TWT measurements did not agree, additional full sets of measurements were obtained until that requirement was fulfilled. Figure 1 illustrates the 3 measurements that were obtained in both sites.

The 22 EUS-MP exams described in this study were performed by senior pediatric GI fellows (RC, BK, DA) under the direction of a single attending (SSR). The fellows performed the EGD and positioned the probe inside of the balloon. The attending added the water, guided placement of the probe, obtained/interpreted the images and recorded the values. Starting in the fall of 2019, the latex balloon replaced the previous procedure of adding free water. After gaining experience with the technique, the additional times required to complete each EUS case were recorded. The aim was to evaluate the feasibility of including EUS, employing the latex balloon and miniprobe, during a standard, diagnostic EGD (Table 1).

**Table 1:** Time to Obtain 12 total esophageal wall and sublayer measurements in 22 consecutive EUS cases

Age	Diagnosis	Time	Data sets required^*^	
			Distal	Mid
7	GER	8:32	2	2
7	EoE-R	7:12	3	2
11	GER	5:34	2	2
12	EoE-A	7:30	2	2
11	EoE-R	4:50	2	2
21	EoE-R	6:01	3	2
15	GER	11:53	2	6^a^
15	EoE-A	10:50	2	3
7	EoE-A	5:02	2	3
21	EGID-A	9:04	2	3
22	EoE-R	8:03	2	2
17	GER	12:51	3	3
18	GER	7:53	2	2
15	EoE-R	6:11	2	2
14	EoE-R	7:31	2	3
24	EoE-A	14:59	2	6^b^
16	EoE-A	9:51	2	2
16	EoE-A	5:16	2	3
15	EoE-A	9:02	3	2
12	EoE-R	6:17	2	2
16	EoE-A	8:09	2	2
16	GER	8:15	2	4^c^

^*^Minimum of 2 sets obtained from both the mid and distal esophagus

^a^Hiatal hernia led to problems defining lower esophageal sphincter

^b^Last study on broken probe

^c^Balloon leakage during procedure

## Results

All cases generated the full set of measurements described above in both the mid and distal esophagus. The table provides the total time to obtain the minimum of 6 measurements from each of the two sites, with each requiring two consistent TWT values as described above. Of the 44 data sets in this series (mid and distal esophagus in the 22 patients), 31 (70%) had the first two TWTs in agreement and only required the minimum two sets. Three patients required > 3 sets of measurements (6, 6, and 4), because of variance in the distal esophageal TWT values. The explanations for additional sets were: a sliding hiatal hernia, a worn-out probe which was replaced after the exam, and difficulties with leakage from the balloon itself. As anticipated, cases which required additional measurements took longer. The average EUS time was 8 min and 13 s. In this small series, even the longest case was completed in 15 min, as noted in the table.

## Discussion

This report describes 22 consecutive pediatric EUS-MP cases that successfully measured the mid and distal esophageal wall layers using a miniprobe inside of a latex balloon sheath filled with water. The series was the recent subset of an IRB approved protocol designed to study EUS in suspected or proven EoE. The protocol design required  two values of the total esophageal wall thickness that had less than 20% variation, and additional measurements of the individual layers in both the mid and distal esophagus. Introduction of the balloon sheath represented a modification of the initial EUS investigation of EoE patients that employed a miniprobe with added water to the esophageal lumen [9]. The balloon permitted 12 or more EUS measurements to be obtained with a total average time of less than 10 min. The modification eliminated the need to sustain adequate free water above the lower esophageal sphincter to obtain sets of duplicate measurements, and the concerns regarding the introduction of free water into the mid esophagus of a sedated child. Thus, the latex sheath around the miniprobe enabled rapid, dependable, safe, and reproducible acoustic coupling.

Presently, diagnostic EUS is primarily employed and promoted as a tool to guide interventions designed to obtain material for subsequent analyses, such as directed biopsies of submucosal lesions [16] or aspiration of pancreatic cysts [17]. The present series illustrates a fundamentally different application of diagnostic EUS, in which precisely obtained measurements of anatomic features are directly employed to recognize and characterize pathology. This is analogous to using echocardiography to investigate cardiac anatomy [18]. Similar to echocardiography, the full potential of this application will require a single universally agreed upon approach for each clinical indication. Ideally, they will be developed and approved by experts in the field and subsequently widely disseminated, as has been proposed for other point of care ultrasound applications [4]. The need for a single standardized method prior to widespread adoption of EUS for clinical and research purposes is highlighted by the measurements of the normal (control group) esophageal TWTs reported in the two earlier EoE series. Although pediatric TWT increases with height and age [15], almost identical TWT values were reported with a EUS-MP in the pediatric (mean age 9 years, 2.1 mm) cohort [9] and an echoendoscope in adults (2.2 mm) [10]. One additional unresolved challenge in this field is interpreting measurements derived through different EUS methodologies. A recent Cochrane review evaluating EUS in gastric cancer characterization concluded that while the technique has clinical value, the heterogeneity of published values needs to be addressed [19].

Nearly two decades after the initial data demonstrating that EUS has potential value in understanding and managing EoE, recent reports have correlated EUS abnormalities with EoE related dysfunction [20, 21]. There is a strong basis to the theory that EUS could potentially improve clinical outcomes in EoE. The present consensus guidelines define remission based on resolution of esophageal eosinophilia and symptoms [22]. However, the morbidity in this disease is based on esophageal remodeling [11] and esophageal remodeling in EoE does not correlate with esophageal eosinophilia [23]. Anecdotal reports have provided intriguing observations regarding the potential role of EUS in defining resolution of remodeling as a clinical end point of EoE therapy [24, 25]. However, recent guidelines on the use of endoscopy in EoE from the American Society of Gastrointestinal Endoscopy [26] and a clinically usable EoE severity scoring system published jointly by gastroenterologists and allergists [27] have both failed to recognize a potential role for EUS in this disease. EUS educational programs providing a background on both EUS technical aspects and ultrasound imaging of the gastrointestinal tract, could enhance widespread employment, as has been seen in POCUS applications in the emergency department [28]. EUS-MP has been the focus of published series from Europe [6] and Asia [13] that each included over 1000 patients.

The major limitation in the present study is that the miniprobe that was utilized is no longer commercially available. Whether the manufacturer will resume distribution is unknown at this time. Secondly, there are no reliable standards to assess the accuracy of the presented esophageal wall dimensions. As previously summarized, published measurements of the esophageal wall have been widely inconsistent [15]. In addition, the reproducibility and validity of this type of approach needs to be established, as the measurments  were obtained by a single endoscopist from a single cohort of patients. Finally, it remains to be established that EUS has a worthwhile role in EoE patient management. While EUS provides detailed *anatomic* features that can track the appearance and reversal of esophageal remodeling, another approach which examines the *consequences* of remodeling is presently being preferentially introduced into the care of EoE patients. ENDOFLIP is a technique that is able to quantitatively evaluate esophageal distensibility, which becomes compromised by the fibrotic changes that occur during remodeling. Whether this tool will be superior to EUS in the management of patients with EoE, or a role for each will emerge, should be the focus of larger studies employing universal guidelines. Recently, transabdominal ultrasound [5, 29] and EUS-MP [30] have been introduced into the care of inflammatory bowel disease, independently demonstrating the capacity of ultrasonography to detect and quantitatively evaluate chronic subepithelial inflammatory/fibrostenotic gastrointestinal wall changes.

## Conclusions

The presented series demonstrates that diagnostic EUS-MP employing a latex sheath to help create the acoustic interface can provide a detailed characterization of the total esophageal wall and its individual layers in a rapid, safe, and cost-efficient manner during routine endoscopy. The series also illustrates that once the endoscopist becomes familiar with the sonographic images of the normal and pathologic gastrointestinal wall and the technical aspects of EUS, a detailed description of any potential subepithelial pathology can be obtained in less than 10 min. This information is then available for immediate discussion with the patient and family, with the potential of increasing both the physician and patient’s satisfaction, as has been described for POCUS [28]. EUS-MP which can be potentially performed during any endoscopic procedure, may thus help the patient avoid the inconvenience of a second radiographic procedure requiring another day off from work, and additional fees from the new physician and facility. The recent position paper of the World Federation for Ultrasound in Medicine concluded that in the future POCUS will be more diverse than ever and be included in medical student training [31]. One unique example could be the routine application of endoscopic POCUS (E-POCUS) to provide comprehensive characterization of any endoscopically observed subepithelial gastrointestinal pathology.

### Supplementary Information

Additional file1. To see the video please go to the online supplementary material.

## Data Availability

The only data in the paper is the time of the procedures which is detailed in the table. The individual EUS measurements will be shared upon request.

## Abbreviations

EUS: Endoscopic ultrasound
EoE: Eosinophilic esophagitis
EGD: Upper endoscopy
GER: Gastroesophageal reflux
TWT: Esophageal total wall thickness
eos/hpf: Eosinophils per high power field
EGID: Eosinophilic gastrointestinal disease
POCUS: Point of care ultrasound
E-POCUS: Endoscopic point of care ultrasound
